# Increasing global temperatures threaten gains in maternal and newborn health in Africa: A review of impacts and an adaptation framework

**DOI:** 10.1002/ijgo.14381

**Published:** 2022-08-16

**Authors:** Matthew F. Chersich, Fiona Scorgie, Veronique Filippi, Stanley Luchters, Climate Change and Heat‐Health Study Group, Andrea Huggett, Euphemia Sibanda, Craig Parker, Darshnika Lakhoo, Gloria Maimela, Helen Rees, Ijeoma Solarin, Lois Harden, Robyn Hetem, Webster Mavhu

**Affiliations:** ^1^ Wits Reproductive Health and HIV Institute (Wits RHI) Faculty of Health Sciences, University of the Witwatersrand Johannesburg South Africa; ^2^ Faculty of Epidemiology and Population Health, London School of Hygiene and Tropical Medicine London UK; ^3^ Centre for Sexual Health and HIV/AIDS Research (CeSHHAR) Harare Zimbabwe

**Keywords:** Africa, climate change, heat, maternal and newborn health, pregnancy, reproductive health, temperature

## Abstract

Anatomical, physiologic, and socio‐cultural changes during pregnancy and childbirth increase vulnerability of women and newborns to high ambient temperatures. Extreme heat can overwhelm thermoregulatory mechanisms in pregnant women, especially during labor, cause dehydration and endocrine dysfunction, and compromise placental function. Clinical sequelae include hypertensive disorders, gestational diabetes, preterm birth, and stillbirth. High ambient temperatures increase rates of infections, and affect health worker performance and healthcare seeking. Rising temperatures with climate change and limited resources heighten concerns. We propose an adaptation framework containing four prongs. First, behavioral changes such as reducing workloads during pregnancy and using low‐cost water sprays. Second, health system interventions encompassing Early Warning Systems centered around existing community‐based outreach; heat‐health indicator tracking; water supplementation and monitoring for heat‐related conditions during labor. Building modifications, passive and active cooling systems, and nature‐based solutions can reduce temperatures in facilities. Lastly, structural interventions and climate financing are critical. The overall package of interventions, ideally selected following cost‐effectiveness and thermal modeling trade‐offs, needs to be co‐designed and co‐delivered with affected communities, and take advantage of existing maternal and child health platforms. Robust‐applied research will set the stage for programs across Africa that target pregnant women. Adequate research and climate financing are now urgent.

## Synopsis

High temperatures increase rates of adverse pregnancy outcomes. Women in Africa are at high risk, and require behavioral, health‐system, built‐environment, and structural interventions.

## 1 BACKGROUND

“As a species, we are expert problem solvers. But we haven't yet applied ourselves to this problem [climate change] with the focus it requires.” Sir David Attenborough, 2019.

### 1.1 Extreme heat in Africa

Africa is the continent hardest hit by climate change, has the fewest resources to respond,^1^ is least responsible for the crisis, and to date has had limited success in securing adequate climate financing and reparations for losses and damages from climate change.^2^ Heat‐health burdens of disease are mounting as the average global temperature rises—it is already 1.2°C above pre‐industrial levels, and a further degree or more warming appears inevitable.^3^ Yet, temperature increases are spread unevenly across the globe. Increases in many parts of Africa are well above the global average.^2^ Heatwaves are becoming more frequent, intense, and longer lasting, and heatwave conditions will occur on at least 50% of days during the warm season in many cities near the Sahel by 2100, assuming the current trajectory of emissions continues.^4^ Already many of the temperatures being recorded in Africa are close to the limits of “liveability”, and physical labor or “workability” is not possible for parts of the year.^5^ Studies in Burkina Faso,^6^ Ghana,^7^ Kenya,^8^ South Africa,^9^ and Tanzania^10^ have shown considerable spikes in overall population‐level mortality on days with higher than average temperatures. Additionally, the number of emergency department visits increased at higher temperatures in Botswana,^11^ as did hospital admissions in Uganda,^12^ and diarrheal rates in children under 5 years of age in South Africa.^7^, ^9^ Studies in Ethiopia, South Africa, and Uganda have documented heat impacts on pregnancy outcomes.^13^, ^14^, ^15^

Many women in Africa have little or no protection against exposure to extreme heat events during pregnancy. Rapid urbanization on the continent means that increasing numbers of pregnant women reside in informal housing in urban heat islands, where temperatures can be several degrees higher than in surrounding areas. Health facilities, poorly built brick dwellings, and informal housing are frequently 4°C–6°C warmer indoors than outdoors.^16^, ^17^ Additionally, in many rural areas in Africa, pregnant women have little or no means of reducing heat exposure, and even drinking water may be scarce or non‐potable.^18^ Levels of knowledge about the harmful effects of high ambient temperatures are low in Africa,^19^ and cultural, social, and religious practices such as outdoor church services, may increase exposure risks.

### 1.2 Pathophysiologic processes and indirect impacts of high ambient temperatures on maternal and newborn health

**FIGURE 1:**
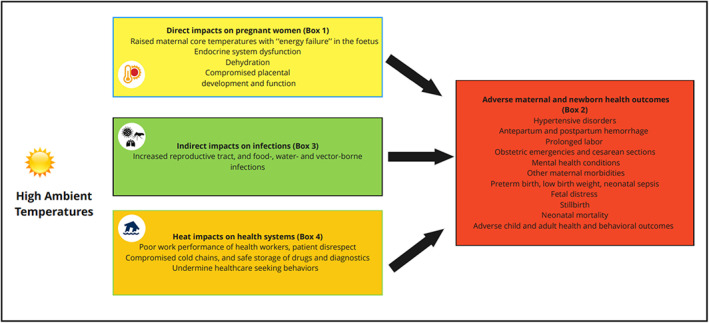
Pathophysiologic processes and indirect impacts of high ambient temperatures on maternal and newborn health.

Worldwide, around 200 studies have documented the negative impacts of exposure to high ambient temperatures on maternal and newborn health.^13^, ^20^, ^21^ The threshold for harmful temperature exposure in pregnant women, however, varies by climate zone, health condition, and individual risk profiles, among other factors. Generally, relationships between temperature and adverse health outcomes are U‐ or J‐shaped, with the base of the exposure‐outcome curve commonly at relatively mild temperatures around 20°C, and the arms of the curves becoming exponentially steeper with each degree increase in temperature or additional day of a heatwave.^13^ Even at relatively mild temperatures, health consequences begin to mount and the harmful impacts of heat exposure are not restricted to heatwaves. The term “extreme heat” is used in this article to denote heat waves and “high ambient temperatures” to encapsulate the full range of temperature patterns that result in adverse health outcomes related to heat, either through direct or indirect pathways. These pathways and their pathophysiologic, infectious disease and health systems mechanisms are delineated below (Figure 1).

High ambient temperatures may overwhelm the capacity of maternal thermoregulatory mechanisms to dissipate heat in pregnancy and labor (Figure 1: Box 1). The physiologic and anatomical changes in pregnancy that challenge the maternal thermoregulation peak in advanced pregnancy and labor, including the considerable endogenous heat generated by fetal metabolism, strain from additional weight gain in pregnancy, fat deposits that retain heat, and the major exertion of labor and childbirth, which generates significant amounts of heat. This endogenous thermal load makes it difficult for pregnant or parturient women to maintain normothermia during periods of high ambient temperatures. We highlight four interlinked pathophysiologic processes that may explain the harmful impacts of heat when thermoregulatory capacity is exceeded during pregnancy or labor.

The first pathway involves elevated maternal body temperatures. In normal labor, maternal temperature increases by about 0.02°C per hour, but at a considerably faster pace among women who are primiparous,^22^ have prolonged labor,^23^ or who are obese.^24^ Additionally, maternal core temperature increase during fever from malaria or other infections, following epidural analgesia,^25^ or if thermoregulatory mechanisms such as perspiration and hyperventilation cannot compensate for the additional burden of endogenous heat during periods with high ambient temperatures. Because fetal temperature is consistently 0.3°C–0.5°C higher than the maternal core temperature,^26^ fetal temperatures can rise to dangerous levels if maternal temperatures increase. A systematic review summing 36 studies showed that maternal hyperthermia around the time of childbirth increased the odds of neonatal brain injuries, such as cerebral palsy, by 2.5‐fold.^27^ Neuronal injury in the fetus related to hyperthermia is ascribed to “energy failure”, where the amount of oxygen and substrates delivered to brain tissue in the fetus are insufficient to meet an increased demand during hyperthermia, leading to brain tissue acidosis, ischemic injury, and long‐term neurologic sequalae in affected children.^28^ In those studies, body temperatures above 39°C in the mother had particularly severe consequences for the fetus, but even mildly elevated maternal temperatures (37.1°C–37.5°C) carried substantial risks for adverse birth outcomes.^29^

Second, maternal dehydration during a heat event can lead to electrolyte imbalances and cause endothelial, oxidative, and inflammatory sequelae,^30^ undermining already burdened cardiovascular and renal systems during pregnancy. Levels of mild dehydration, as measured by urine specific gravity, can reach 40% among women during pregnancy and childbirth, even during periods with normal temperatures.^31^, ^32^ Within the fetus, dehydration can manifest as severe oligohydramnios during extreme heat events.^33^ Third, high ambient temperatures can interrupt endocrine system function, increasing oxidative stress, inflammation, and the release of heat‐shock proteins, which affect placental adaptation to hypoxia, cell death regulation, and cytoskeletal rearrangements.^34^, ^35^ Extreme heat events in pregnancy have been linked with abnormal glucose tolerance and gestational diabetes mellitus, both of which can negatively affect maternal and newborn health.^36^ At high ambient temperatures, adrenaline and stress hormones rise, adversely affecting mental health and well‐being, of concern given the already heightened risk for mental health conditions during pregnancy and postpartum.^37^, ^38^ Lastly, in a fourth mechanism, heat stress affects placental growth, development, and function.^39^ Chronic thermal stress is associated with “placental shrinkage” or reduction in volume and weight of the placenta, as shown in human^39^ and animal^40^ studies. This limits placental transport efficiency, including for oxygen and nutrients. During more acute periods of thermal stress, shunting of blood to the body surface reduces uterine blood flow and causes placental hypoperfusion.^41^

The four interlinked pathophysiologic processes described above manifest clinically in a broad spectrum of conditions (Figure 1). Maternal health conditions linked with high ambient temperatures include hypertensive disorders of pregnancy (e.g., pre‐eclampsia and eclampsia),^42^, ^43^ including in a recent study in South Africa,^14^ preterm prelabor rupture of membranes,^44^ placental abruption,^45^ antenatal and postpartum hemorrhage,^42^, ^43^ renal disease,^43^ and increased rates of emergency cesarean sections^24^, ^43^ (Figure 1: Box 2). Data are not yet available on the impacts of high ambient temperatures on maternal deaths, though this is likely as heat exposure increases, the risk for all the principal causes of maternal deaths. Less frequent causes of these deaths in Africa also appear heat sensitive, for example, in one study in the USA the risk of a cardiovascular event during childbirth rose by 11% with each 1°C increase in temperature.^46^

Heat exposure can lead to increased uterine contractions,^47^, ^48^ fetal tachycardia and reduced fetal movements, as demonstrated in studies among pregnant women exposed to high temperatures in thermal chambers^49^ or through intrauterine temperature monitors in women during labor.^50^ Clinically, during periods of high ambient temperatures there are increased rates of fetal distress,^42^ preterm birth (increased in a meta‐analysis by 1.16‐fold during heatwaves, for example),^13^ low birth weight,^13^ stillbirths (increased in a meta‐analysis by 1.24‐fold at high versus low temperatures),^13^ meconium aspiration,^42^ neonatal jaundice,^51^ neonatal intensive care admissions,^52^ and perinatal mortality (increased 1.53‐fold in one study when temperature exceeded the 95th centile).^53^ Furthermore, several studies, including in Ethiopia^54^ and Uganda,^15^ have suggested that exposure to high ambient temperatures in utero affects health throughout the life course.^55^

Indirect impacts of high ambient temperatures include increased incidence of food‐, water‐ and vector‐borne infections (Figure 1: Box 3). High temperatures promote replication and survival of microbes and disease vectors. Infections of the genital tract, such as group B streptococcus,^56^ which is the leading cause of bacterial pneumonia, sepsis, and meningitis in neonates, are especially concerning, in particular if labor is prolonged during heat extremes. Malaria infections and shifts in distribution to areas with non‐immune populations have major implications for maternal health as pregnant women are at high risk for complicated malaria.^5^, ^57^

High ambient temperatures place additional strains on health systems, including through reducing work performance of health providers (Figure 1: Box 4).^58^ Further, levels of irritability and anxiety among health workers may rise as temperatures escalate,^38^ potentially leading to patient disrespect. Extreme heat events threaten cold chains, and the safe storage of drugs and diagnostics. A study in Malawi showed that drug storage temperatures exceeded the recommended 25°C–30°C threshold for oxytocin in half of the 40 facilities assessed.^59^ In another study in France, a large spike in cases of postpartum hemorrhage was ascribed to damage to an oxytocin batch during a heatwave, and rates of postpartum hemorrhage returned to baseline once a new batch of oxytocin was received.^60^ High ambient temperatures may also undermine healthcare seeking behaviors during pregnancy.^61^ Many women wait in long queues to receive antenatal care, which is challenging during hot periods. Moreover, though no evidence is available, it is possible that during labor the burden of traveling by foot or in crowded, non‐ventilated vehicles to facilities during periods of high ambient temperatures may make some women favor home over facility birth. Women giving birth under the care of a traditional birth attendant or a family member may lack protections during periods with high ambient temperatures: a study in Bangladesh, for example, found that newborns born at home during periods of extreme heat were 1.14‐fold more likely to have neonatal illnesses (e.g., serious infections) than those born at home on cooler days.^62^ Additionally, periods of high ambient temperatures, accompanied by soil drying, can reduce crop and livestock yields, raising risk for malnutrition and reducing income for pregnant women. In turn, reduced income may limit a woman's ability to meet the out‐of‐pocket costs of accessing maternity care.

Taken together, it is clear that the escalations in temperatures owing to climate change will have major consequences for maternal and child health in Africa, and progressively so as temperatures escalate. Establishing large‐scale programs to address these burdens is a major priority, but requires a robust evidence base to inform funding allocations. In the section that follows we present an approach to interventional research and program design that has been developed by the Climate Change and Health Group.

## 2 AN APPROACH TO HEAT‐HEALTH RESEARCH AND PROGRAMMING FOR SECURING MATERNAL AND NEWBORN HEALTH

**FIGURE 2:**
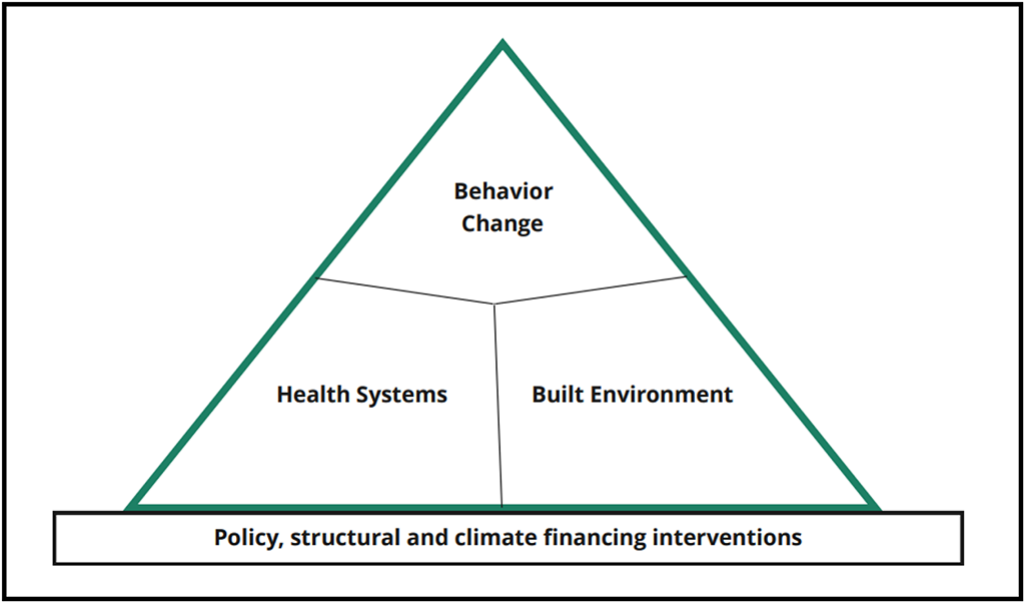
Framework for reducing impacts of high ambient temperatures on maternal and newborn health.

In recent decades, it has become apparent that, with adequate intervention, health risks from extreme heat can be ameliorated. Within many high‐income countries, heat‐related mortality and morbidity have declined with successive heatwaves as adaptation measures have improved, approaching zero excess deaths in some areas.^5^, ^63^ These declines may be attributable to interventions encompassing four domains, namely (1) behavioral changes; (2) health system inputs, including the development of new health services; (3) changes to the built environment, in particular air conditioning^64^, ^65^, ^66^; and (4) structural and policy interventions (Figure 2). We describe the salient features of these domains below, and how they relate to maternal and newborn health.^67^

### 2.1 Behavior change interventions

Information campaigns and behavior change interventions that target pregnant women, female family members (e.g., mothers‐in‐law), male partners, community leaders, and other stakeholders could support self‐care interventions during pregnancy.^68^ Such interventions include maintaining hydration during hot periods, accessing cool areas, or seeking shade; using low‐cost water sprays; wearing appropriate clothing, ideally cotton; and self‐monitoring for symptoms of heat illness, especially dizziness, heavy sweating, fatigue, and clammy skin with goosebumps.^64^, ^65^, ^66^ Many pregnant women continue to do physical work even late in gestation, including walking long distances to collect water and firewood. Interventions are needed to shift these workloads to other family members during pregnancy. Promotion of hydration and water supplementation during periods of high ambient temperatures, such as in “Water‐Rest‐Shade” initiatives, can have major benefits.^69^

Coverage of antenatal care and community‐based services are high in many parts of Africa, meaning that pregnant women have frequent contacts with the health system, providing multiple opportunities for reinforcement of behavior changes. Several mHealth initiatives have specifically targeted pregnant women (e.g., MomConnect SMS program in South Africa^70^ and Wired Mothers in Zanzibar, Kenya^71^), offering an additional medium for heat‐health messaging. Further, preparedness and complication readiness—key tenets of heat planning responses—have been at the heart of health promotion in maternal health, and could be adapted to encompass heat‐related interventions. Birth preparedness, for example, includes helping women to plan in advance for their transport during labor,^72^, ^73^ which may be particularly important during periods of high ambient temperatures.

### 2.2 Health system and service interventions

The considerable investments in Africa in infrastructure, services, and human resources for maternal health provide a platform for interventions such as Early Warning Systems. Warning systems form the mainstay of heat‐health services in high‐income countries. Here, when weather forecasts predict that a pre‐specified temperature threshold will be exceeded, warnings are issued and a series of tiered interventions are triggered. Then, when the heat event occurs, health services focus on protecting high‐risk groups, including pregnant women and infants.^64^, ^65^, ^66^ This light include home “check‐ups” of pregnant women by community health workers, and facilitating access to “cooling areas” in the community or in a maternity facility. Heightened monitoring during childbirth for potential complications of heat exposure is prudent, such as more frequent assessments of water intake and hydration status, labor duration, blood loss, and signs of infection, especially group B streptococcus. In hot climates, water supplementation, even in people with very mild dehydration, can have considerable positive impacts.^74^ Other health system interventions could include integrating indicators within the district health systems to track the burden of heat‐related conditions in pregnant women and newborns, and ensuring that the cold chain for drugs is maintained during extreme heat events, especially for oxytocin.

### 2.3 Built environment, cooling, and nature‐based solutions

**FIGURE 3:**
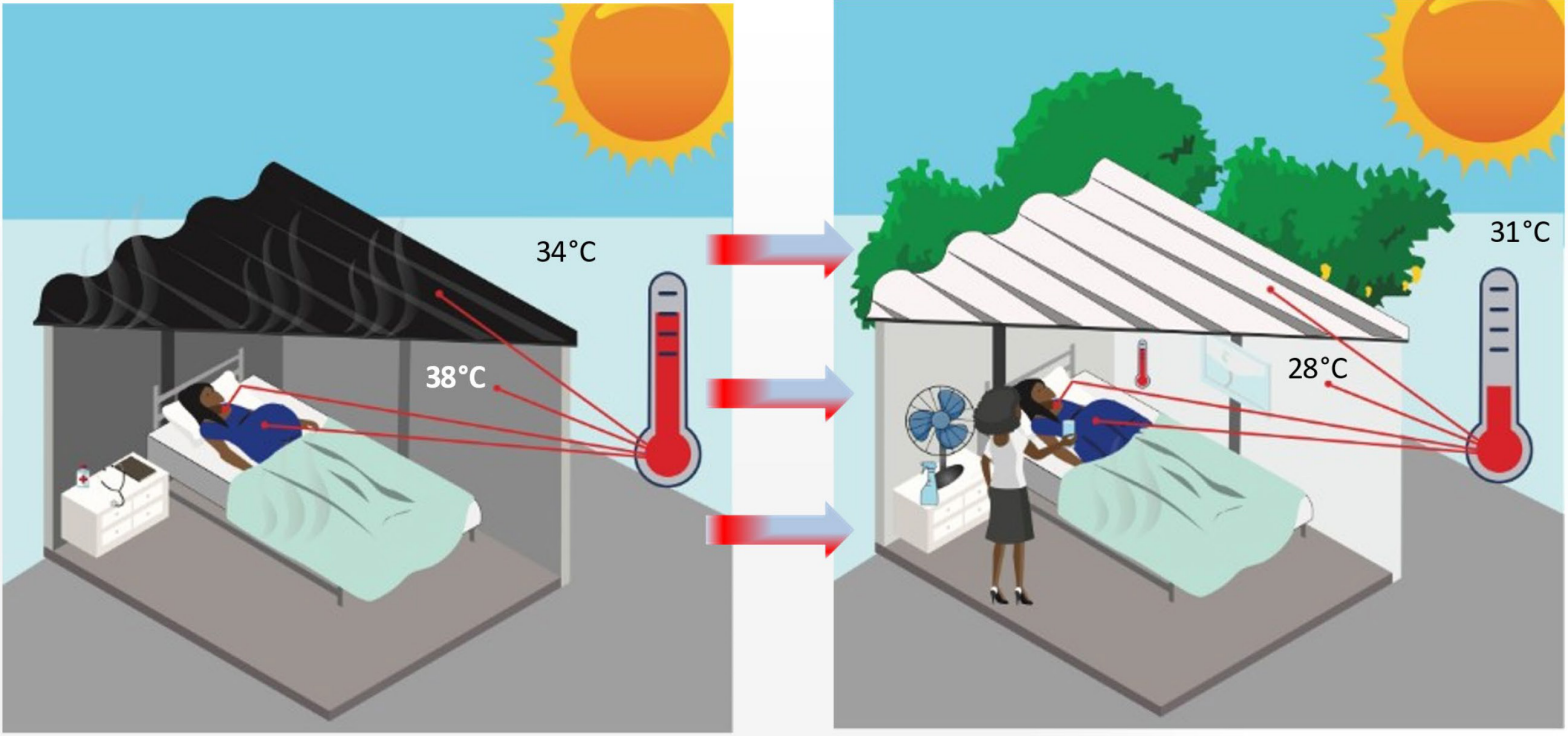
Potential built environment, cooling and nature‐based solutions.

Most women in Africa give birth in a health facility, meaning that providing an environment for childbirth that protects pregnant women and newborns against high ambient temperatures may have a high impact (Figure 3). Passive cooling can provide high levels of sustained protection. This may include minor modifications to labor wards to alter interactions between a building envelope and the natural elements. Simple facade fixes such as awnings, overhangs, louvres, and insulated walls and roofs are highly effective at reducing solar radiation. “Heat proofing” includes enhancing heat resistance of buildings through increasing reflectivity of surfaces (enhanced albedo) and the provison of localized solutions such as mist sprays and evaporative materials. White reflective paint for rooftops can reflect almost all solar radiation.^66^

Indigenous knowledge around building design and urban planning holds much promise, with, for example, traditional earthen housing construction with straw roof materials providing substantial thermal comfort advantages over poorly insulated concrete housing and sheet iron roofs. Natural ventilation strategies such as chimneys and air vents (e.g., “whirly birds”) in homes and maternity wards could facilitate cool air circulation and dissipate heat. The effectiveness of passive cooling, however, varies widely, depending on the location of the sun and wind direction, among other factors. Careful thermal modeling simulations and cost‐effectiveness evaluations can assist in selecting the optimum building modifications.

Air conditioning is referred to as “tantamount to a potentially life‐saving medical device” for groups with high heat vulnerability.^66^ But, equally, the widespread use of air conditioning is “a clear example of maladaptation to climate change”. Hence, notwithstanding the multiple drawbacks of air conditioning (e.g., using as much as 8.5% of total electricity consumption and more than half of electricity on hot days, high energy costs, noise pollution, heat generation, power outages, and its signaling of widening global “thermal inequities”),^5^ if used appropriately, it may have an important role in protecting maternal and newborn health. Use of air conditioning in a few designated “cool spaces”, such as labor wards, can provide protection during extreme heat events and may be cost‐effective. There are direct parallels between the concept of Maternity Waiting Homes, which serve to shelter and protect pregnant women, and the notion of having dedicated cooling “shelters” in maternity hospitals. Many lessons from waiting homes around acceptability, recipient selection, and implementation may also be applicable to “cooling centres”.^72^ Maintaining comfortable temperatures in facilities would also help to reduce thermal discomfort for maternity health workers, shoring up their performance, and potentially averting irritability or aggressive behaviors. Electric and hand‐held fans with light water spraying offer low‐cost, potentially scalable alternatives to air conditioning.^5^

Nature‐based “green and blue” solutions around maternity facilities and at the homes of pregnant women can reduce temperatures by several degrees, and are often low cost and considered an essential part of “bottom‐up” community‐led adaptations. Interventions may include street trees, green roofs, green walls, ponds and other water features, where feasible. Shading from tree canopies reduces radiant heat loads, a key determinant of outdoor thermal comfort around antenatal clinics, for example. Moreover, trees and other vegetation produce considerable cooling from evapotranspiration. Greening also has several environmental and health co‐benefits, including noise reduction, improvements in social well‐being, and amelioration of the mental health impacts of high ambient temperatures.

### 2.4 Structural, policy, and climate financing interventions

Major investments are needed to improve the social and economic conditions that underlie much of the vulnerability to climate change in Africa. Most especially, many of the factors that place pregnant women in Africa at risk during periods of high ambient temperatures relate to resource constraints, and the deficiencies in housing, nutrition, and social services that mark the continent. Resilience to climate shocks is low in most parts of Africa. Intense, prolonged heat waves can have devastating impacts on crop and livestock resources, placing pregnant women and newborns at high risk for malnutrition. Rates of metabolism are raised markedly during periods of high temperatures, heightening nutritional needs. Clearly, pregnant women need to be a high priority population for food support. Cash transfers provided to pregnant women have multiple benefits for pregnant women and newborns, aside from improving food security.^75^ Of major concern, few countries in Africa have developed policies around climate change and health. The CHANCE network established within the EU Horizons ENBEL project aims to address this key gap. A conducive policy environment is essential for developing programs and services around behavioral, health systems, and the built environment as described above.

Commitments to provide adequate climate financing were a central part of the Paris Accord and subsequent meetings of global leaders. Scant progress, however, has been made to meeting these commitments. For example, only a small fraction of the US$ 100 billion per year promised by high‐income countries has been provided.^76^ Climate financing earmarked for enhancing resilience in pregnant women and for programming to provide heat‐related services for pregnant women may go a long way towards addressing the growing burden of heat‐related conditions among pregnant women in Africa.

## 3 RESEARCH AND PROGRAMMATIC PRIORITIES IN AFRICA

Climate change threatens to undo the hard‐fought gains made by maternal and child health programs in Africa over the past few decades.^77^ These gains are fragile: maternal and newborn mortality in Africa remains the highest worldwide, with two out of every three maternal deaths in the world occurring in sub‐Saharan Africa for example,^78^ and rates of preterm birth in Africa remain the highest globally.^79^ Clearly, adaptation interventions that result in even small reductions, for example, risk for preterm births, will have major cost savings and lifelong benefits for affected individuals.

A cluster of factors and health system opportunities make pregnant women a highly suitable target group for heat‐related interventions in Africa. Considerable investments in shoring up maternal and child health services—at both facility and community level—on the continent means that these services provide an ideal platform for heat‐health interventions. Indeed, maternal and child health services form the cornerstone of primary health care in Africa and have proven historically to be adept at incorporating new programmatic areas, as shown by HIV mother‐to‐child transmission programs.^80^

What is needed are intervention packages that draw on a range of behavioral, health systems, and built environment solutions, optimized in each setting. These packages need to be systematically co‐designed with pregnant and postpartum women, health workers, male partners, and other stakeholders. Qualitative, ethnographic research, and participatory co‐design processes are key for tailoring heat adaptation interventions to different socio‐cultural and geographical contexts. Selection of the package of interventions requires careful consideration of the cost‐effectiveness trade‐offs between potential interventions. Integrating heat‐related interventions within existing programs for maternal and newborn health may enhance sustainability in the long run. Importantly, many health workers do not yet have the knowledge and skills required to protect pregnant women and newborns against high ambient temperatures, and training is needed, both pre‐ and in‐service.

Evidence from rigorous studies is required to ensure that climate financing aid to assist African countries to adapt to climate change is used optimally. The limited research to date on climate change adaptation in Africa, however, means that there are major gaps in evidence. Research among pregnant women is the ideal place to begin building an evidence base, which can then be extended to other vulnerable groups on the continent. To our knowledge, worldwide, only one study has so far set out to examine heat adaptation interventions in pregnant women (the CHAMNHA study, funded by the Belmont Forum).^81^ In fact, globally, across all population groups, most assessments to date of effectiveness of heat adaptation interventions have been based on post‐hoc analyses of trends in health surveillance data after heat waves.^52^, ^63^

Clearly, formulating, testing, costing, and scaling up solutions to protect pregnant women against high ambient temperatures is a major public health priority. There is also a moral imperative to do so: women in Africa have made a negligible contribution to global carbon emissions, yet may be among the populations most affected by climate change and have the fewest resources to cope, especially while pregnant. Large‐scale research projects among pregnant women in Africa and then heat‐heath programs at scale are long overdue.

## AUTHOR CONTRIBUTIONS

MFC, FS, VL and SL contributed to the conceptualization of the paper, writing of drafts of the articles and preparation of the final manuscript. Climate Change and Heat‐Health Study Group members contributed to the draft writing and editing of the manuscript.

## CONFLICT OF INTEREST

MFC and FS hold investments in the fossil fuel industry through their pension funds as obligated by the Wits Health Consortium. The University of the Witwatersrand holds investments in the fossil fuel industry through their endowments and other financial reserves. No other conflicts of interest declared.

## DATA AVALILIBILITY STATEMENT

Data sharing is not applicable to this article as no new data were created or analyzed in this study.

## Appendix

CLIMATE CHANGE AND HEAT‐HEALTH STUDY GROUP

Andrea Huggett (Wits RHI), Euphemia Sibanda (CeSHHAR), Craig Parker (Wits RHI), Darshnika Lakhoo (Wits RHI), Gloria Maimela (Wits RHI), Helen Rees (Wits RHI), Ijeoma Solarin (Wits RHI), Lois Harden (Department of Physiology, Faculty of Health Sciences, University of the Witwatersrand, Johannesburg, South Africa), Robyn Hetem (School of Animal, Plant and Environmental Sciences, Faculty of Sciences, University of the Witwatersrand, Johannesburg, South Africa), and Webster Mavhu (CeSHHAR).
